# The Feasibility and Performance of Thin-Film Thermocouples in Measuring Insulated Gate Bipolar Transistor Temperatures in New Energy Electric Drives

**DOI:** 10.3390/mi16040465

**Published:** 2025-04-14

**Authors:** Bole Xiang, Guoqiang Li, Zhihui Liu

**Affiliations:** 1 School of Optoelectronic Engineering, Chongqing University of Posts and Telecommunications, Chongqing 400065, China; 2021210537@stu.cqupt.edu.cn (B.X.); 2021210848@stu.cqupt.edu.cn (G.L.); 2 State Key Laboratory of Tribology in Advanced Equipment, Tsinghua University, Beijing 100084, China

**Keywords:** IGBT, thin-film thermocouple, chip temperature measurement field, switching frequency, duty cycle

## Abstract

In the new energy electric drive system, the thermal stability of IGBT, a core power device, significantly impacts the system’s overall performance. Accurate IGBT temperature measurement is crucial, but traditional methods face limitations in IGBT’s compact working space. Thin-film thermocouples, with their thin and light features, offer a new solution. In this study, Ni 90% Cr 10% and Ni 97% Si 3% thin-film thermocouples were prepared on polyimide substrates via magnetron sputtering. After calibration, the Seebeck coefficient of the thin-film thermocouple temperature sensors reached 40.23 μV/°C, and the repeatability error stabilized at about 0.3% as the temperature rose, showing good stability. Researchers studied factors affecting IGBT temperature. Thin-film thermocouples can accurately monitor IGBT module surface temperature under different conditions. Compared to K-type wire thermocouples, they measure slightly higher temperatures. As the control signal’s switching frequency increases, IGBT temperature first rises then falls; as the duty cycle increases, the temperature keeps rising. This is consistent with RAC’s junction temperature prediction theory, validating the feasibility of thin-film thermocouples for IGBT chip temperature measurement. Thin-film thermocouples have great application potential in power device temperature measurement and may be a key research direction, supporting the optimization and upgrading of new energy electric drive systems.

## 1. Introduction

In the current era of global automotive intelligence, with the burgeoning demand for automotive chips, insulated gate bipolar transistor (IGBT) chips, as the core components of power conversion systems [1,2], have emerged as one of the key elements in new-energy vehicles, accounting for 7–10% of the total vehicle cost. Amid the variable working environments and complex operating conditions, IGBTs are significantly influenced by fluctuations in junction temperature [3,4]. Consequently, real-time measurement and control of the junction temperature of IGBT power devices are of utmost importance for enhancing the reliability of power conversion systems.

Thin-film thermocouples, which are extensively applied in surface temperature measurement, possess numerous advantages. They can measure both static and dynamic temperatures and directly output DC voltage signals. Their micron-scale dimensions are well-suited to the narrow working spaces of equipment [5,6], and they feature high-temperature measurement accuracy, small heat capacity, and short response times. These attributes endow thin-film thermocouples with distinct advantages in signaling, automatic recording, and automatic control [7,8,9,10].

In 2020, Liu, Xingliang et al. [11] measured the temperature of an IGBT module using the eddy-current pulse thermography method. In the same year, Humphrey Mokom Njawah Achiri et al. [12] employed the Least Squares Principle to determine an alternative IGBT thermal impedance network. Y. Zhang [13] proposed a physical contact thermometry method for evaluating the junction temperature distribution inside a pressure-packed IGBT in 2020. Also in 2020, Tang, Y. et al. [14] measured the junction temperature in real-time using a high-speed infrared thermography device. In 2021, Lim, H. et al. [15] analyzed the junction temperature by sampling the NTC temperature of IGBT modules. J. Mai et al. [16] measured the surface temperature of IGBTs using three methods: fiber-optic grating (FBG), thermocouple, and infrared thermography, in 2021. Dou, Y. et al. [17] proposed a back-propagation (BP) neural network prediction model to obtain accurate IGBT junction temperature in 2021. In 2023, Arya, Abhinav et al. [18] proposed a Junction Temperature Estimation Technique to address errors in the thermal mode measurement of IGBT temperatures. Tong, An et al. [19] from the Beijing Institute of Technology predicted the IGBT junction temperature by modeling the Cauer thermal network of the IGBT module in 2023.

Based on the above-mentioned research, it has been found that there are four primary methods for measuring the junction temperature of devices: infrared temperature measurement, the NTC temperature-sensitive electric parameter method, the thermal network modeling method, and the contact temperature measurement method. Although all these methods are widely utilized in various encapsulation forms, each has certain limitations.

Infrared thermometry can cause some structural damage to the package. It requires the removal of the package shell, the dissolution of silica gel, and the spraying of the temperature-measurement area with black paint to ensure uniform thermal emissivity. In contrast to the infrared temperature-measurement method, within the IGBT package module, manufacturers often integrate an NTC temperature sensor for IGBT temperature monitoring, obviating the need for costly infrared equipment. However, the time constant of the NTC sensor is much larger than the temperature-rising rate of the IGBT wafer, making it impossible to directly protect the IGBT wafer by monitoring the NTC temperature.

Regarding thermal network modeling, effective results can only be achieved within a limited scope. It is challenging to account for temperature-related variables under transient conditions (such as wide-range environmental temperature changes) or during overload or short-circuit operations [20]. Moreover, the more complex the thermal resistance model is, the more difficult it becomes to implement the real-time estimation algorithm for the inverter junction temperature.

The contact temperature-measurement method can accurately measure the device’s temperature distribution in real-time by contacting the sensor with the device. However, with the miniaturization trend in the microelectronics industry, device modules are becoming smaller. This requires careful consideration of the sensor’s volume, posing certain difficulties in integrating the sensor into the device.

To meet these challenges, we propose a novel solution: the thin-film thermocouple sensor. This sensor has typical two-dimensional characteristics, with thermal junction thicknesses on the order of micro-nanometers. Compared to ordinary thermocouple sensors, they offer advantages such as small size, small heat capacity, fast response, high sensitivity, and ease of integration. Employing thin-film thermocouples in the field of IGBT device temperature measurement can effectively address the issues of miniaturization and response speed. On this basis, a new thin-film thermocouple sensor system was established to comprehensively analyze the temperature of IGBT power equipment. This system is not only accurate and reliable, but also meets the requirements of miniaturized devices, providing crucial support for the real-time junction temperature control of IGBT power devices.

## 2. Thin-Film Thermocouple Temperature Sensor

### 2.1. Principles of Thin-Film Thermocouple

**Figure 1:**
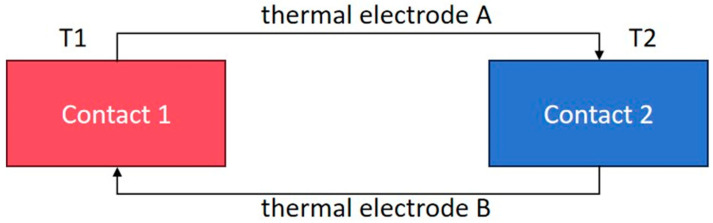
Schematic diagram of the thermoelectric effect.

A thin-film thermocouple consists of two distinct conductive materials, denoted as A and B, which form a closed loop. As depicted in Figure 1, the temperature is detected via contacts 1 and 2. The thermoelectric effect gives rise to the potential at these two contacts. Contact 1 serves as the measuring end, also known as the hot end, while contact 2 functions as the compensation end, or the cold end, which is maintained at a constant temperature of 0 for compensation purposes.

Owing to the fact that the two contacts are at different temperatures, a temperature-difference potential exists between hot electrode A and hot electrode B. In this closed-loop structure, a current is generated, and electrons move from the high-temperature region to the low-temperature region. Consequently, the total voltage within the circuit is the combination of the contact potential and the temperature-difference potential, which is precisely the manifestation of the Seebeck effect [21].

The potential difference between the conducting materials A and B is

(1) $$∆V=S_{AB}\left(T_{2}-T_{1}\right)=S_{AB}∆T$$

The relative Seebeck coefficient is

(2) $$S_{AB}=\underset{∆T\to 0}{\lim}\frac{∆V}{∆T}=\frac{d∆V}{d∆T}$$

where $S_{AB}$ represent the Seebeck coefficients of the two different materials A and B themselves, and T_1_ and T_2_ are the temperatures at the combination of contacts 1 and 2.

### 2.2. Thin-Film Thermocouple Temperature Sensor Preparation

#### 2.2.1. Experimental Platform Construction

Thin-film thermocouple temperature sensors are mainly composed of components such as the substrate, insulating film, thermocouple material, silicon oxide film, conductive silver glue, and compensation wire.

The initial step is substrate selection. Commonly used substrates include sapphire, ceramics, polyimide, etc. In this experiment, the required substrate material should be small-sized, have a low thermal expansion coefficient, be able to maintain stable chemical properties at high temperatures above 150 °C, and be able to adhere closely to the chip surface. A comparison of commonly used substrate materials, as presented in Table 1, was carried out. Eventually, the polyimide film (PI film) was chosen as the flexible substrate material for this experiment. It has an extremely low thermal expansion coefficient, ranging from 2 × 10^−5^ to 10 × 10^−5^/°C.

Simultaneously, for the IGBT power device with temperature measurement in this paper, an insulating film can serve as a dielectric layer for interlayer insulation. It not only enhances the bonding force between the thermocouple material and the substrate, but also prevents the diffusion of metal elements on the surface of the IGBT power device into the thermocouple film, which could otherwise affect the performance of the thin-film thermocouple (TFTC). Taking into account the insulating performance and the volume of the insulating film, the thickness of the aluminum oxide in the insulating film is controlled to be 1000 nm.

Subsequently, the thermocouple material is prepared. Based on previous research, the NiCr/NiSi material is selected, with a NiCr ratio of 90:10 and a NiSi ratio of 97:3 [22]. Previous studies have shown that as the thickness of the NiCr/NiSi film increases from 400 nm to 2000 nm, the Seebeck coefficient of NiCr/NiSi TFTC increases [10]. Therefore, in this article, through co-sputtering, the thickness is controlled to be 900 nm. The measurable temperature range of the prepared K-couple encompasses the temperature range of IGBT power devices, and features advantages such as high linearity, sensitivity, large thermoelectric potential, and low cost [23]. As a result, the NiCr/NiSi material is chosen as the functional thin-film material in this paper, and the temperature of IGBTs is measured using the TFTC.

During the magnetron sputtering process, to form the hot electrode of the thermocouple, it is necessary to cover the mask plate to achieve this objective.

**Figure 2:**
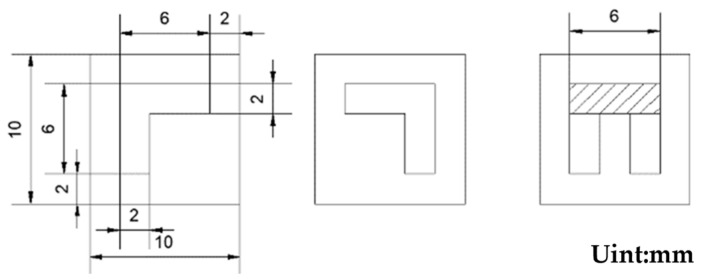
Masking board design drawing.

**Figure 3:**
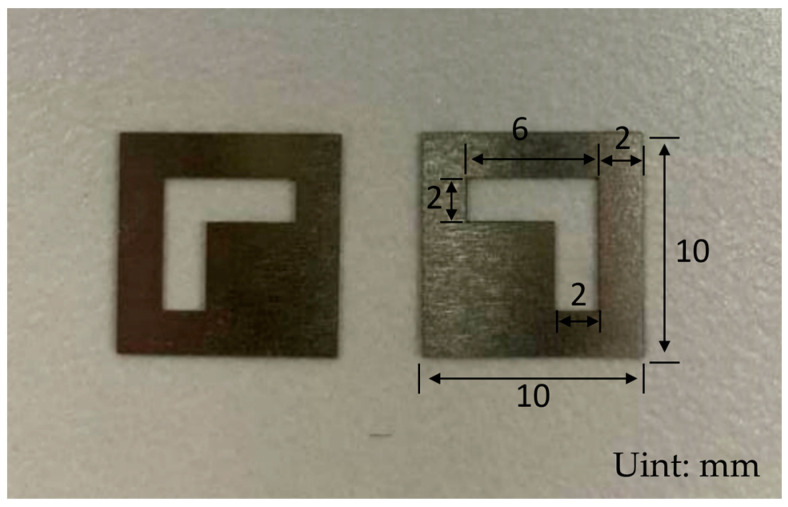
Masking board physical picture.

As shown in Figure 2, this experimental design mask plate has an overall length of 10 mm, width of 10 mm; the hollow sputtering part is 7-shaped, 6 mm long, 6 mm wide; the hollow part is located in the center of the mask plate; the boundary from the edge of the mask plate is 2 mm, and the preparation of the physical structure is as shown in Figure 3.

#### 2.2.2. Thin-Film Thermocouple Temperature Sensor Preparation Process

When preparing a TFTC, the following steps are carried out. Initially, an alumina insulating film is deposited. Subsequently, mask plate 1 is placed over the insulating film, and magnetron sputtering is conducted using a NiCr target. Once the sputtering with the NiCr target is finished, mask plate 2 is placed over the insulating film already sputtered with NiCr, and magnetron sputtering is then carried out using a NiSi target.

**Figure 4:**
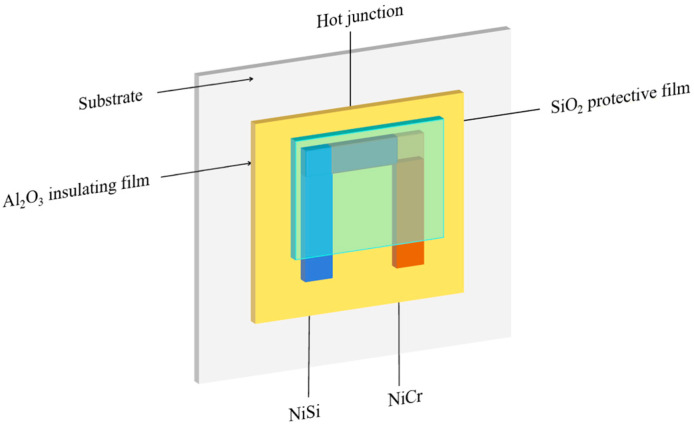
Thin-film thermocouple temperature sensor schematic.

The mask plates are designed in such a way that an overlap region is retained. This overlap region contains both NiCr and NiSi, which forms the thermal contact of the TFTC. Finally, a 2000 nm thick silicon oxide film is deposited over the structure. This silicon oxide film serves a dual-purpose function: it acts as an isolation layer and provides protection for the underlying components [24,25]. The designed NiCr-NiSi TFTC is shown in Figure 4.

The preparation process of the compensation line for the TFTC temperature sensor is as follows. First, the insulation films at both ends of a 15 cm NiCr compensation line and a 15 cm NiSi compensation line were removed. Then, the resistance values of the NiCr and NiSi compensation lines were measured separately using a First Source DM 3068 six and a half digit digital multimeter (RIGOL Technologies, Co. Ltd. Beijing, China). The results indicated that the resistance value of the NiCr compensation line was 4.076 Ω, while that of the NiSi compensation line was 2.705 Ω. The parameters for NiCr and NiSi thin film deposition are shown in Table 2.

**Figure 5:**
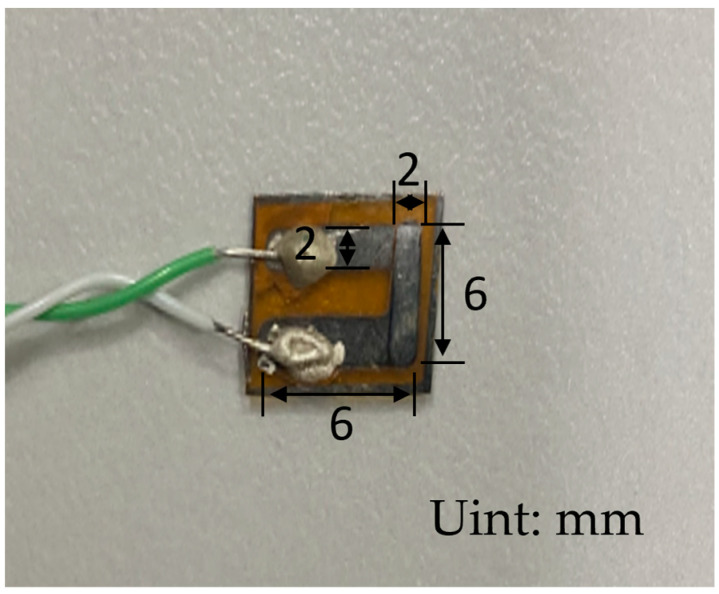
Thin-film thermocouple temperature sensor physical drawing.

After the resistance measurement, the two compensation lines were fixed on the desktop with polyimide tape, keeping an appropriate distance between them. Next, the compensation leads of the TFTC temperature sensor were connected to the thin film using conductive silver adhesive. After the conductive silver gel had rested and solidified for 10 h, the TFTC temperature sensor was successfully prepared. The final product is presented in Figure 5.

### 2.3. Thin-Film Thermocouple Performance Studies

In order to test the accuracy of the output signals of the developed TFTC temperature sensor and the commercially proven K-type wire thermocouple in the same environment, a static calibration was carried out. The static calibration system mainly includes heat source equipment and collection equipment. The heat source equipment is used to provide a stable temperature field for the thermocouples, and is also selected to provide a stable room temperature as the cold end for thermocouple temperature compensation. The acquisition equipment is then used to collect the thermal potential generated by the thermocouple in the loop. In order to ensure accuracy and comparability, the K-type wire thermocouple were static calibrated along with self-prepared film thermocouples.

To assess the accuracy of the output signals of the newly developed TFTC temperature sensor and the commercially validated K-type wire thermocouple within the same environment, a static calibration procedure was conducted. The static calibration system primarily consists of heat source equipment and data acquisition equipment. The heat source equipment serves to create a stable temperature field for the thermocouples, and also provides a consistent room temperature to act as the cold end for compensating the thermocouple temperatures. Subsequently, the acquisition equipment is utilized to gather the thermal potential generated within the thermocouple loop. To ensure precision and comparability, the K-type wire thermocouple was calibrated statically alongside the self-made TFTC.

1. Heat source equipment.

In this study, the HANBANG electronic HP-E1515 constant temperature heating station was employed as the hot end of the static calibration system. Capable of setting temperatures up to 400 °C, this heating station can directly achieve intelligent and stable temperature control via its internal CPU. It also offers an intuitive display of the set temperature. Moreover, its user-friendly and convenient operation streamlines the calibration process, and make it more efficient.

2. Acquisition equipment.

The RIGOL DM3068 six-and-a-half-digit desktop digital multimeter (RIGOL Technologies, Co. Ltd. Beijing, China) was selected as the acquisition device for measuring the thermal potential of the TFTC. Renowned for its high precision, it boasts a maximum measurement rate of 10 Krdgs/s. Integrating functions such as automatic measurement and various digital conversions, it is both easy to operate and highly flexible.

**Figure 6:**
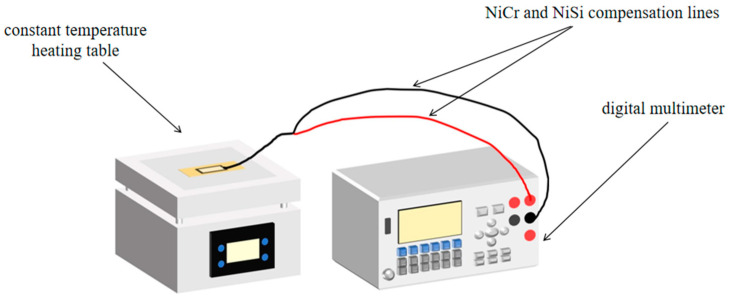
Static calibration system connection diagram for thin-film thermocouple and K-type wire thermocouple temperature sensors.

Specifically, as illustrated in Figure 6, the hot end of the thermocouple was attached to the center of the thermostatic heating table, while the cold end was placed directly in the air at a constant temperature. The digital multimeter was then used to record the thermal potential. To obtain a more reliable Seebeck coefficient for the TFTC, the temperature range of the heat source constant temperature heating table was set from 50 °C to 400 °C. The cold end was maintained at room temperature. Thus, the static calibration temperature was calculated as the temperature of the constant temperature heating table minus the room temperature. With the current room temperature at 15 °C, the actual static calibration temperature range was from 35 °C to 385 °C. To ensure temperature stability, the temperature of the constant temperature heating table was allowed to stabilize for 5 min for every 5 °C increase. The thermal potential was then recorded at different static calibration temperatures. Given that the commercial K-type wire thermocouple, due to its tape encapsulation, can only measure temperatures up to a maximum of 215 °C, the actual static calibration temperature range for this thermocouple was adjusted to be from 35 °C to 200 °C.

Based on the above principle of Seebeck effect, the expression for temperature versus voltage during static calibration is

(3) E=Sθ+B

**Figure 7:**
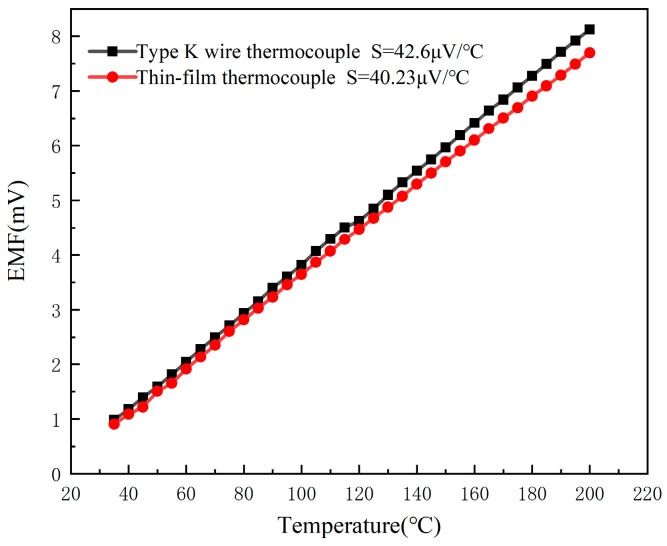
Static calibration curves for K-type wire thermocouple and thin-film thermocouple temperature sensors.

E describes the thermopotential of the thermocouple. θ is the measured temperature. S denotes the Seebeck coefficient, which represents the voltage generated per degree Celsius of temperature difference. B is the intercept term used to correct for the baseline voltage offset at 0 °C. The static calibration thermopotential–temperature relationship for the K-type wire thermocouple and the developed TFTC is shown in Figure 7. The data generated from the static calibration experiments were analyzed by Origin 2018 analysis software, and the static calibration curves, fitting equations, correlation coefficients, and nonlinear fitting errors for the developed K-type wire thermocouple and the TFTC are derived by using the least squares method. The fitting equation for the output thermopotential E of a K-type wire thermocouple for temperature measurement to the static calibration temperature is: E=0.0426θ−0.45326, and the Seebeck coefficient S is 42.6 μV/°C. The fitting equation for the output thermopotential E of the TFTC temperature measurement to the static calibration temperature is: E=0.04023θ−0.34948, the Seebeck coefficient S is 40.23 μV/°C. Although the Seebeck coefficients of the two are close, the value of the TFTC is slightly smaller than that of the K-type wire thermocouple. This difference is mainly due to the fact that electrons within the positive NiCr thin film in the TFTC can only enter the NiSi thin film through the overlapping region of the thin film. Due to the limitation of film thickness, the positive film cannot provide enough free electrons to effectively transition to the negative film, so at the same temperature, the Seebeck coefficient of the TFTC is less than that of the K-type wire thermocouple [23].

**Figure 8:**
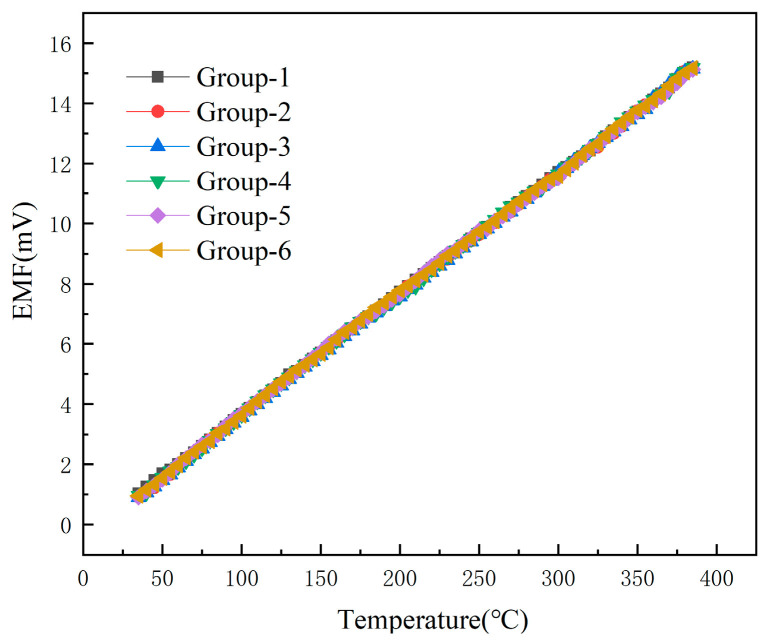
Thin-film thermocouple temperature sensor repeatability calibration curve.

In order to test the accuracy and repeatability of the output signals of the developed TFTC temperature sensor and K-type wire thermocouple in the same environment, the repeatability test of the TFTC was made on the basis of the above static calibration. The same TFTC was tested for six times by using the above static calibration method, and the curves of these six repetitions are shown in Figure 8.

The data from the above six experiments were analyzed for repeatability error and the standard deviation was calculated as

(4) $$\sigma =\sqrt{\frac{\sum {\left(X_{i}-X\right)}^{2}}{n-1}}$$

where n represents the actual number of measurements, $X_{i}$ is the result of each measurement, X is the average of multiple measurements and σ is the standard deviation.

After calculating the standard deviation, calculate the repeatability error based on this to evaluate the stability of the film thermocouple. The repeatability error is calculated as

(5) $$\delta =\frac{\sigma }{x}\times 100\%$$

where δ is the repeatability error. x is the average of multiple measurements and σ is the standard deviation.

**Figure 9:**
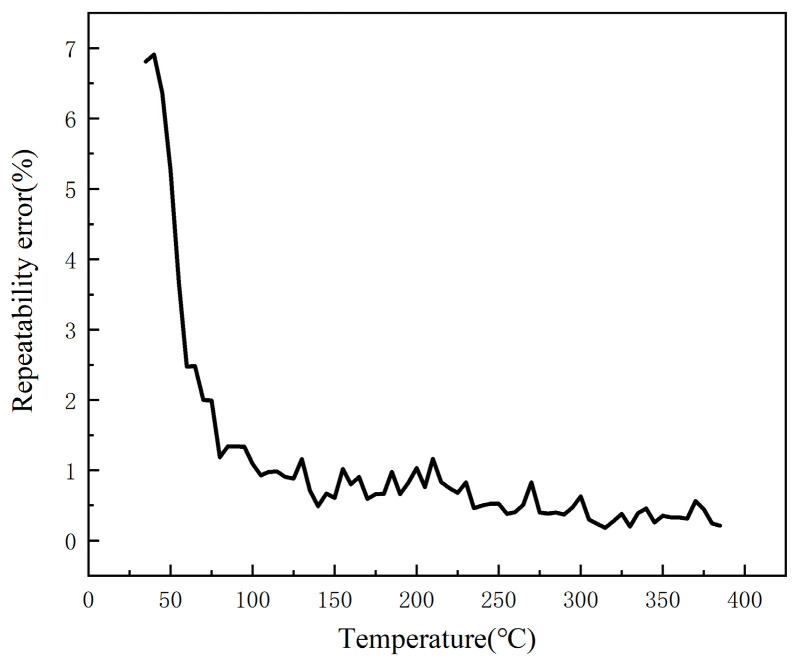
Repeatability error of thin-film thermocouple temperature sensors at different temperatures.

In Figure 9, it can be seen that, when the temperature is 35 °C, the sensor repeatability error is the largest, 6.81%, with the gradual increase in temperature, the repeatability error gradually decreases, and finally stabilized at 0.3%.

In conclusion, the developed film thermocouple Seebeck coefficient is large, high temperature measurement accuracy, error within a reasonable range, good repeatability, can be realized in the use of the process of repeated accurate temperature measurement.

### 2.4. Introduction to IGBT Modules

SGT40N6ONPFDPN insulated gate bipolar transistor (40N60IGBT) using a new generation of field stop (Field Stop) process, is a modular semiconductor products from the insulated gate bipolar transistor and FWD (current–supply diode chip) through the specific circuit bridging package, with a low on-state loss and switching loss, a positive temperature coefficient is easy for parallel applications. The low conduction loss and switching loss, positive temperature coefficient and easy parallel application are some of the features.

The 40N60IGBT power electronic module of the overall loss generated by the insulated gate bipolar transistor and the FWD of their respective losses and, therefore, in the work of the cause of its heating there are three reasons, one is the conduction of the on-state loss (Ploss_on), the second is the switching loss generated in the process of turning on and off, and the third is the clogging loss, but due to the clogging loss is relatively small, usually can be ignored [26]. This paper focuses on the pass-state loss and switching loss of IGBT modules. Due to its own on-state voltage drop is not zero, so the pass-state loss is generated, IGBT unit pass-state loss formula is

(6) $$P_{CT}=I_{cav}\times V_{ce0}+R_{c}\times I_{crms}^{2}$$

(7) $$I_{cav}=D\times I_{o}$$

(8) $$I_{crms}=D\times I_{0}^{2}$$

where $V_{ce0}$ is the zero-current conduction state collector-emitter voltage, $R_{c}$ is the collector-emitter resistance, $I_{cav}$ is the average value of the current, $I_{crms}$ is the rms value of the current, $I_{o}$ is the output current, and D is the duty cycle.

The IGBT cell switching loss equation is

(9) $$P_{SWT}=f_{SW}\times (E_{onM}+E_{offM})$$

where $f_{SW}$ is the switching frequency; $E_{on}$ is the turn-on loss; $E_{off}$ is the turn-off loss.

For the FWD chip, the pass-state power consumption is

(10) $$P_{CD}=I_{Dav}\times V_{D0}+R_{D}\times I_{Drms}^{2}$$

(11) $$I_{Dav}=\left(1-D\right)*I_{o}$$

(12) $$I_{Drms}=\left(1-D\right)*I_{0}^{2}$$

where $V_{D0}$ is the on-state voltage drop, $R_{D}$ is the on-state resistance, $I_{Dav}$ is the average value of current, $I_{Drms}$ is the rms value of current, $I_{o}$ is the output current, and D is the duty cycle.

The switching power consumption is

(13) $$P_{SWD}=f_{SW}\times E_{onD}$$

where $f_{SW}$ is the switching frequency, $E_{onD}$ is the turn-on loss.

The total power consumption of the IGBT chip is

(14) $$P_{T}=P_{CT}+P_{SWT}$$

The total power consumption of the FWD chip is

(15) $$P_{D}=P_{CD}+P_{SWD}$$

The total loss of the 40N60 IGBT power electronics module is equal to the sum of the total power consumption of the IGBT chip and the total power consumption of the FWD chip, which is

(16) $$P=P_{T}+P_{D}$$

**Figure 10:**
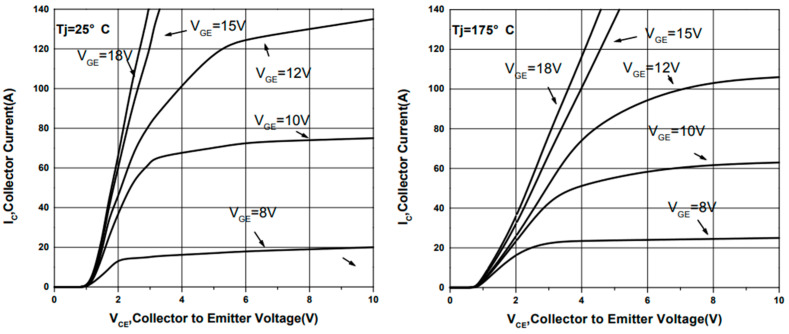
Typical output curve for 40N60IGBT.

$R_{D}$ and $R_{c}$ and the voltages can be obtained by consulting the 40N60IGBT chip manual and combining them with the operating currents, as shown in Figure 10.

As a high-power semiconductor device, the overall performance and reliability of IGBT modules are influenced by temperature. From a macro point of view, is simply due to the high power loss, IGBT will produce more heat. From a micro perspective, the essence of heat generation is the reduction in kinetic energy of electrons during flow, which is lost in the form of heat energy. Given that the junction temperature of the IGBT cannot exceed 175 °C, and should not be operated at higher temperatures for a long period of time, temperature monitoring of the IGBT module is essential. In order to realize the temperature monitoring of the IGBT module, in this paper, the above calibrated film thermocouple and silk thermocouple are laminated on the surface of 40N60IGBT in TO-247 package. By allowing the 40N60IGBT to function properly in the circuit, these two thermocouples together form a thin-film thermocouple-based IGBT chip surface temperature test system. When actually monitoring the junction temperature, it is necessary to combine the circuit, power consumption and thermal resistance to calculate the accurate junction temperature in the chip.

### 2.5. Construction of IGBT Module Temperature Test System Based on Thin-Film Thermocouple

The 40N60IGBT temperature measurement system mainly includes: a 40N60IGBT working system, film thermocouple, K-type wire thermocouple, and voltage acquisition equipment.

1. The 40N60IGBT operating system.

**Figure 11:**
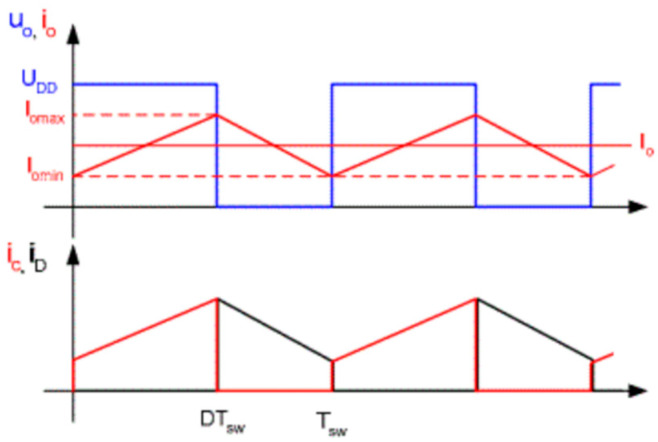
Typical signals for a single-quadrant purely resistive load drive.

In this paper, the test 40N60IGBT operating system is through the microcontroller output control signals, switching load circuits, and in the middle of adding optocoupler isolation circuit to prevent interference. The entire load circuit is powered by a 12 V lithium polymer battery, and the output is directly connected to the load, which has Ainuo DC electronic load AN23103H devices and gold aluminum case high-power resistors; for different experimental variables, you need to adjust different output loads, easy to observe and set the output power and output current. For the experimental setup, a typical signal for a purely resistive load driver module is shown in Figure 11. The curve in the figure is represented by the logo of the same color in the upper left corner of the central axis.

2. Temperature measuring film.

**Figure 12:**
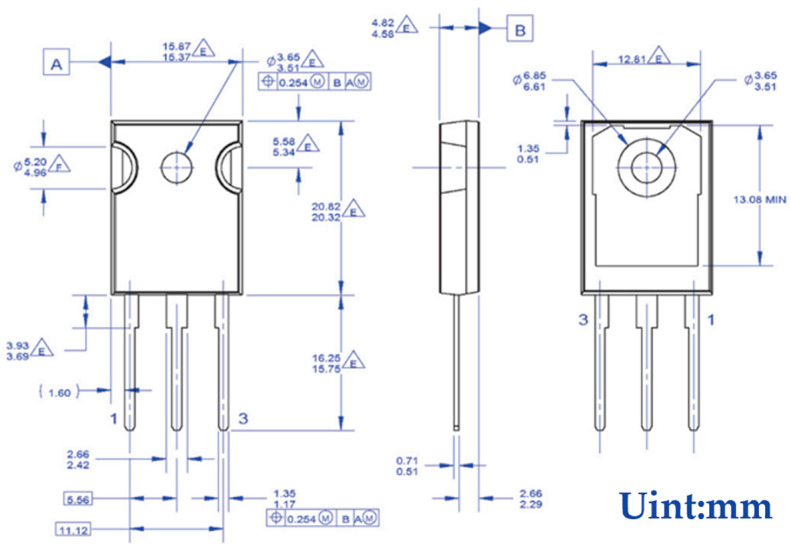
40N60IGBT package outline diagram.

The SGT40N6ONPFDPN is packaged in a TO-247 package with specific dimensions, as shown in Figure 12. The length and width are larger than those of the self-designed film thermocouple, which makes it easy to stick the film thermocouple on its surface. During the experiment, the self-designed and calibrated film thermocouples and K-type wire thermocouples are pasted on the surface of the 40N60IGBT, which is convenient to accurately sense the temperature change.

3. Voltage acquisition equipment.

The selected Puyuan DM3068 six and a half desktop digital multimeter is close to the surface of the IGBT module of the film thermocouple temperature measurement of the thermal potential of the acquisition of equipment, and calibration experiments with the use of equipment consistent with the acquisition of the trigger form is set to the external trigger; the input of a 2 Hz square wave was attached to the external trigger pin of the multimeter, and we set the delay time of 0.5 s to collect the voltage once.

### 2.6. Experimental Operation of Temperature Measurement of IGBT Module Based on Thin-Film Thermocouple

**Figure 13:**
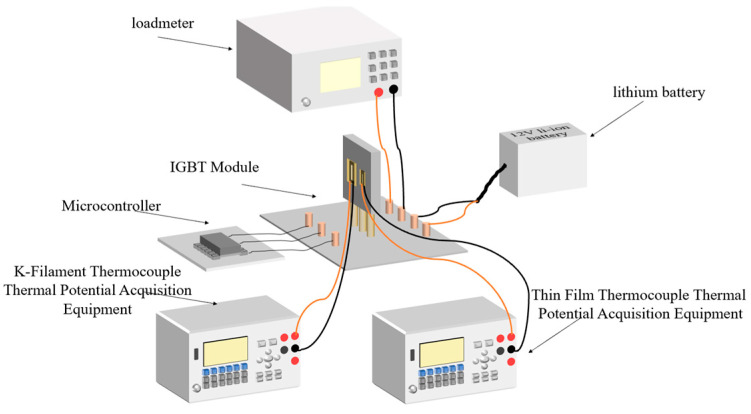
Connection diagram of IGBT module temperature testing system based on thin-film thermocouple and K-type wire thermocouple.

The temperature measurement system we designed is shown in Figure 13. The self-designed and calibrated film thermocouple and K-type wire thermocouple are affixed to the surface of the 40N60IGBT, the microcontroller output control signals to control the circuit operating state, the 12 V lithium battery accesses the load circuit, and at the same time, the digital multimeter measures the film thermocouple and filament thermocouple, substituting for its temperature in the process of static calibration and the voltage expression, to derive the current temperature. From the above Formulas (6) and (10) can be obtained that the pass-state loss is proportional to the operating current, so in the analysis of the pass-state loss, set the control signal is normally open, the load access Ainuo DC electronic load AN23103H equipment, while considering the heat dissipation situation, set the output current and the two variables of the heat sink; from the Formulas (9) and (13) can be obtained that the switching power consumption is related to the switching frequency, and the pass-state loss is related to the duty cycle. Therefore, when analyzing the overall loss of 40N60IGBT power electronic module, the load is connected to a 5-ohm high-power resistor, and the switching frequency and duty cycle are used as variables [27] to analyze the warming rate, cooling rate, and equilibrium temperature of the IGBT in different situations.

## 3. Experimental Data Analysis

### 3.1. Differences in Temperature Measurement Between Thin-Film Thermocouple and K-Type Wire Thermocouple

**Figure 14:**
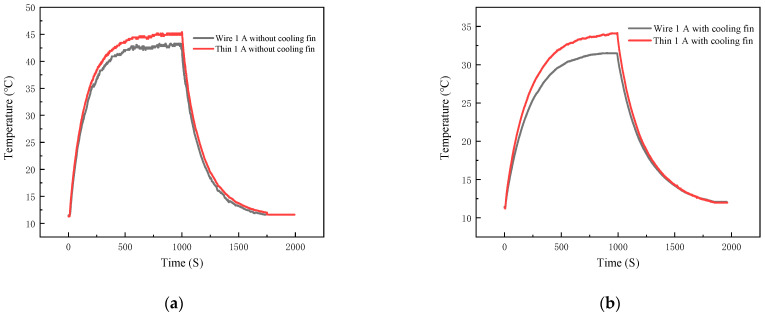
emperature profiles of thin-film thermocouple and K-type wire thermocouples under group 1 and group 2 conditions: (**a**) under group 1 conditions; (**b**) under group 2 conditions.

**Figure 15:**
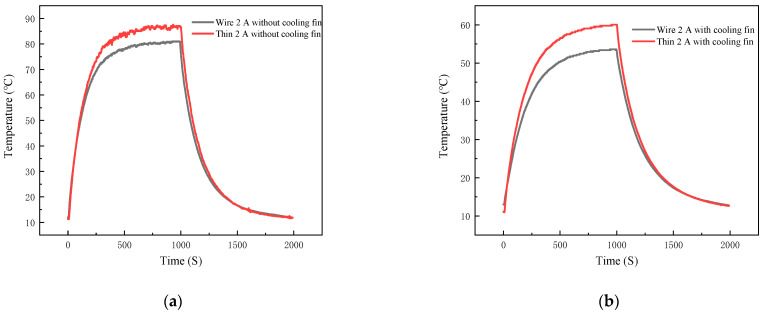
Temperature profiles of thin-film thermocouple and K-type wire thermocouples under group 3 and group 4 conditions: (**a**) under group 3 conditions; (**b**) under group 4 conditions.

First, a detailed comparison is made regarding the temperature measurement differences between the TFTC and the K-type wire thermocouple. Two variables, namely the heat sink and the output power, are set up to conduct four sets of experiments. These experiments aim to measure the temperature of the IGBT module under the following conditions: with a load power of 11.68 W and without a heat sink (Group 1), with a load power of 11.68 W and with a heat sink (Group 2), with a load power of 21.68 W and without a heat sink (Group 3), and with a load power of 21.68 W and with a heat sink (Group 4). The temperature measurement curves obtained from these four sets of experiments for both the TFTC and the K-type wire thermocouple are illustrated in Figure 14 and Figure 15.

As shown in Figure 14a, when the power supply is switched on, a 1 A current flows through the 40N60IGBT. Since the on-state voltage drop of the 40N60IGBT itself is non-zero, an on-state loss occurs, causing the 40N60IGBT to start heating up. As the operating time of the 40N60IGBT increases, the temperatures measured by both the TFTC and the K-type wire thermocouple keep rising. The maximum temperature rise rate measured by the TFTC, which is 0.0778 °C/S, is notably higher than that measured by the K-type wire thermocouple (0.0757 °C/S). This is because the TFTC has a small heat capacity and a rapid response time. However, both thermocouples reach thermal equilibrium at 555 s. At this moment, the rate of heat generation due to passive losses is equal to the rate of passive convective heat dissipation, enabling the chip’s temperature to achieve dynamic equilibrium. The equilibrium temperature measured by the TFTC is 45.15 °C, while that measured by the K-type wire thermocouple is 42.80 °C, resulting in a temperature difference of 2.35 °C. This temperature disparity can be attributed to the difference in their Seebeck coefficients. Specifically, the Seebeck coefficient of the TFTC (40.23 μV/°C) is smaller than that of the K-type wire thermocouple (42.6 μV/°C). According to the Seebeck coefficient formula, the temperature measured by the TFTC is thus higher than that measured by the K-type wire thermocouple.

At the 1000 s mark, the power supply is turned off. Consequently, no current flows through the 40N60IGBT, and heat generation ceases. The air, acting as a cooling agent through passive convection, causes the temperature to start declining. Over the subsequent period, the temperatures measured by both the TFTC and the K-type wire thermocouple continue to drop. The maximum cooling rate of the 40N60IGBT measured by the TFTC, which is 0.0505 °C/S, is significantly greater than that measured by the K-type wire thermocouple (0.0464 °C/S). This difference in cooling rates is similar to the difference observed in the warming rates. Eventually, both thermocouples register the room temperature at 1742 s.

In Figure 14b and Figure 15a,b, the temperature-curve trends of the TFTC and the K-type wire thermocouple are similar to those in Figure 14a. The specific data are presented in Table 3. From the four sets of experimental conditions, it can be observed that the equilibrium temperature, the maximum warming rate, and the maximum cooling rate measured by the TFTC are all higher than those measured by the K-type wire thermocouple. Moreover, the larger the equilibrium temperature, the greater the difference between the maximum warming rate and the maximum cooling rate measured by the two thermocouples.

### 3.2. Effect of Heat Sink on Temperature

Next, analyze the effect of the heat sink on the temperature; the working principle of the heat sink is based on the principle of heat transfer, that is, the transfer of heat must rely on heat-conducting materials and heat transfer medium. The heat sink itself is made of thermally conductive metal, which is attached to the surface of the chip, and so on 40N60IGBT heat is transferred to it, and through a higher surface area, the heat is transferred to the environment.

The common chip cooling path is as follows [28]:

Step 1: heat from the wafer is thermally transferred to the package housing.

Step 2: then, from the package, heat conduction to the surrounding air flow, air due to the temperature difference occurs passive convection.

The path of heat dissipation after adding a heat sink is as follows:

Step 1: heat from the wafer is transferred to the package housing.

Step 2: heat transfer from the package to the heat sink.

Step 3: the heat on the heat sink heat conduction to the surrounding air flow, air due to the temperature difference occurs passive convection.

The basic formula for convective heat transfer in the last step is the Newtonian cooling equation.

(17) Φ=hA∆T

where Φ is the convective heat dissipation per unit time, h is the surface heat transfer coefficient, A is the effective convective heat dissipation area, and ∆T is the temperature difference between the convective heat dissipation surface and the fluid.

**Figure 16:**
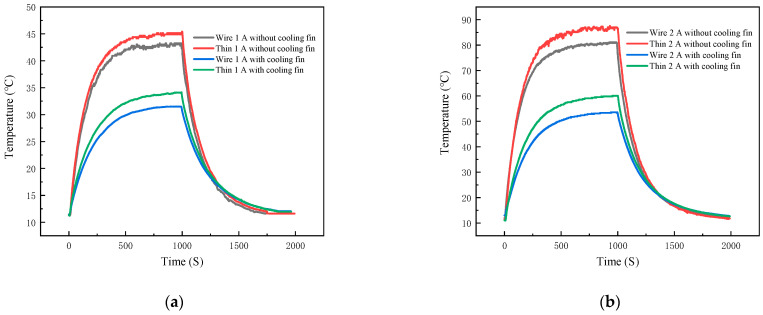
Thin-film thermocouple and K-type wire thermocouple temperature curves at output 1 A and output 2 A: (**a**) output 1 A; (**b**) output 2 A.

For this heat sink on the temperature of the experiment, film thermocouples and K-type wire thermocouple temperature measurement curve as shown in Figure 16a, 40N60IGBT with a load of 21.68 W, Figure 16b, 40N60IGBT with a load of 11.68 W. The above figure shows that both TFTC and k-wire thermocouples are in good agreement in tracking the dynamic temperature distribution of the 40N60IGBT module under different loads. Under 21.68 W load, the equilibrium temperature and heating rate of the film thermocouple are reduced by 31.6% and 52.2%, respectively. The cooling rate was also reduced by 48.7%, confirming the enhanced thermal stability. At 11.68 W load, the equilibrium temperature is reduced by 25.4%, and the heating and cooling rates are reduced by 50% and 23%, respectively. Detailed data are shown in Table 4. The ability of thin film thermocouples to accurately capture these trends is consistent with the Newton cooling principle, where the increase in convective surface area increases the efficiency of heat dissipation.

### 3.3. Effect of IGBT Output Current on Temperature

**Figure 17:**
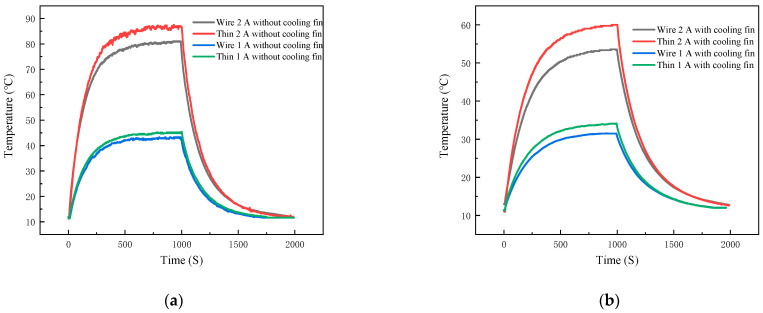
Temperature profiles of Thin-film thermocouple and K-type wire thermocouples without heat sink and with heat sink: (**a**) without heat sink; (**b**) with heat sink.

Next, the effect of 40N60IGBT output current on temperature is discussed. The temperature measurement curves of film thermocouples and K-type wire thermocouple are shown in Figure 17, Figure 17a shows 40N60IGBTs without heat sink, and Figure 17b shows 40N60IGBTs with heat sink. Detailed data are shown in Table 5. From the first paragraph of the analysis can be seen whether it is a film thermocouple or K-type wire thermocouple, the difference between the trend of the temperature of the measurement of no effect, so here to film thermocouple temperature measured as an example. The experimental data in Figure 17 show that increasing the output current from 1 A to 2 A significantly improves the thermal dynamics of the 40N60 IGBT module, regardless of the presence of a heat sink. For the module without heat sink, the equilibrium temperature is increased by 41.91 °C, the maximum heating rate is increased by a factor of 2.9, and the cooling rate is increased by a factor of 2.6 when the output current is doubled. This is consistent with the conduction loss equation $P_{{loss}_{on}}=I_{c}\times V_{ces}$, where higher currents amplify power dissipation and therefore require greater convective heat transfer to reach equilibrium. Thus, the larger temperature gradient drives a faster cooling rate.

With the heat sink, the output current doubles, the equilibrium temperature increases by 25.87 °C, the heating rate increases by a factor of 2.7, and the cooling rate increases by a factor of 1.8. Despite the heat sink’s improved convective heat dissipation efficiency at high loads, the equilibrium temperature at 2 A is still higher than the 1 A configuration without the heat sink, indicating that the heat sink’s capacity is not sufficient to fully offset the losses induced by the doubled current.

### 3.4. Effect of Switching Frequency on Temperature

The switching mechanism of IGBT is exactly the same as that of VDMOS, with the MOS gate controlling its turn-on and turn-off. The difference is that the IGBT than VDMOS in the drain more than a PN junction, in the conduction process there are a few holes involved, which is known as the conductance modulation effect. This effect makes the on-state voltage drop of IGBT lower than that of VDMOS at the same withstand voltage. Due to the presence of holes in the drift region, these holes must disappear from the drift region when the IGBT is turned off, and it takes a longer time to turn off the IGBT device compared to a VDMOS multiplet device. The effect of long switching time on losses is also reflected in the following experiments.

**Figure 18:**
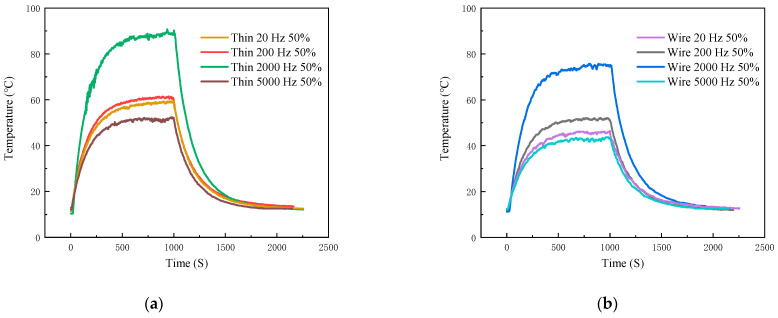
Thin-film thermocouple and type K-type wire thermocouple temperature curve in different frequency: (**a**) thin-film thermocouple; (**b**) type K-type wire thermocouple.

Next, we discuss the impact of the 40N60IGBT switching frequency on temperature. In this experiment, a 5-ohm high-power resistor is connected as the output load, and the output current remains constant. The control switching frequencies are set at 20 Hz, 200 Hz, 2000 Hz, and 5000 Hz, respectively, while the duty cycle is controlled at 50%. The temperature-measurement curve obtained by the TFTC is shown in Figure 18a, and that of the K-type wire thermocouple is shown in Figure 18b. Detailed data are shown in Table 6. From Figure 18, it can be observed that whether using a TFTC or a K-type wire thermocouple, the type of thermocouple has no impact on the temperature-measurement trend. Thus, we take the temperature measurement of the TFTC as an example for analysis.

The specific data are shown in the table below. At 2000 Hz, the IGBT has a maximum heating rate of 0.1735 °C/s and an equilibrium temperature of 89.01 °C, which greatly exceeds the values at 200 Hz and 20 Hz. However, at 5000 Hz, the heating rate drops to 0.0845 °C/s with an equilibrium temperature of 52.09 °C, which is attributed to the shortened effective conduction time due to gate delay, thus limiting the accumulation of switching losses. This is consistent with the theoretical modeling of switching losses, where the heating rate and the equilibrium temperature of the IGBT increase proportionally with increasing switching frequency until a critical frequency threshold is reached. Beyond this critical frequency threshold, parasitic effects such as gate delay shorten the effective conduction time, which leads to a decrease in the heat generation rate and equilibrium temperature, i.e., the loss initially increases with frequency, but decreases under conditions of excessively high frequency due to unfinished switching cycles. Meanwhile, the cooling rate is directly related to the equilibrium temperature gradient, i.e., Formula (17). The higher the steady state temperature, the faster the cooling rate due to the enhanced convective heat transfer caused by the large temperature difference. The reliability of TFTC for high-frequency power electronics was verified by temperature-dynamic experiments of 40N60IGBTs at different switching frequencies, ranging from 20 Hz to 5000 Hz.

### 3.5. Effect of Duty Cycle on Temperature

**Figure 19:**
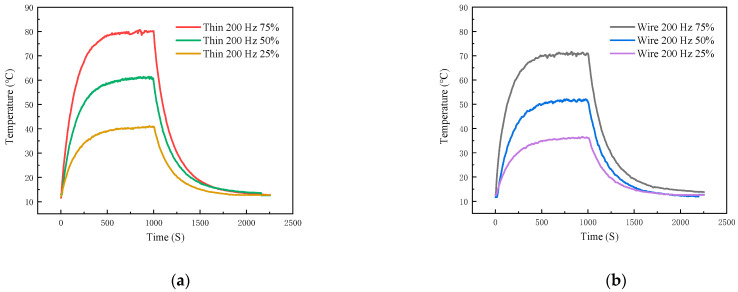
Thin-film thermocouple and K-type wire thermocouple temperature curves in different duty: (**a**) thin-film thermocouple; (**b**) type K-type wire thermocouple.

Next, we explore the influence of the 40N60IGBT control-signal duty cycle on temperature. In this experiment, a 5 Ω high-power resistor is connected as the output load, and the output current remains constant. The control switching frequency is set at 200 Hz, and the duty cycles are set at 25%, 50%, and 75%, respectively. The temperature-measurement curves obtained by the TFTC and K-type wire thermocouple are shown in Figure 19a,b. Detailed data are shown in Table 7. As analyzed in the previous section, whether using a TFTC or a K-type wire thermocouple, the type of thermocouple has no impact on the temperature-measurement trend. Thus, we take the temperature measurement of the TFTC as an example for further analysis.

The maximum warming rate of the 40N60IGBT with a control-signal duty cycle of 75% is 0.1753, which is greater than that with a duty cycle of 50% (0.1271 °C/S). The maximum warming rate of the 40N60IGBT with a duty cycle of 50% is greater than that with a duty cycle of 25% (0.0701 °C/S). The equilibrium temperature of the 40N60IGBT with a control-signal duty cycle of 75% is 80.06°, the equilibrium temperature with a duty cycle of 50% is 60.98°, and the equilibrium temperature with a duty cycle of 25% is 40.72 degrees. Evidently, both the maximum warming rate and the equilibrium temperature increase as the duty cycle increases.

According to Equations (6)–(16), as the duty cycle increases, the root-mean-square (RMS) value of the IGBT chip current rises, while the RMS value of the FWD chip current drops. So, when the frequency is fixed, the temperature of the IGBT chip increases with the growth of the duty cycle, and the temperature of the FWD chip decreases. However, since the collector-emitter resistance of the IGBT chip is larger than the on-resistance of the FWD chip, the increase in the IGBT chip’s temperature has a more significant impact on the overall module. Therefore, at a certain frequency, the overall module temperature rises as the duty cycle increases, which is consistent with the actual experimental results.

The maximum cooling rate of the 40N60IGBT with a control-signal duty cycle of 75% is 0.2378, which is greater than that with a duty cycle of 50% (0.1604 °C/S). The maximum cooling rate of the 40N60IGBT with a duty cycle of 50% is greater than that with a duty cycle of 25% (0.1017 °C/S). This is also because the higher the equilibrium temperature, the larger the temperature difference, and thus the greater the cooling rate.

## 4. Conclusions

In this research, magnetron sputtering technology was employed to fabricate a Ni 90% Cr 10% and Ni 97% Si 3% TFTC. The Seebeck coefficient of the fabricated TFTC was calibrated to be 40.23 μV/°C, while that of the purchased K-type wire thermocouple was calibrated to 42.60 μV/°C. Evidently, the Seebeck coefficient of the developed TFTC is comparable to that of the purchased K-type wire thermocouple. This indicates that the developed TFTC has a Seebeck coefficient close to that of the K-type wire thermocouple, ensuring high-accuracy temperature measurement. Through repeatability experiments, it has been demonstrated that the temperature-measurement error of the TFTC lies within a reasonable range. As the temperature rises, the repeatability error gradually decreases and eventually stabilizes at 0.3%. This implies excellent repeatability, enabling accurate repeated temperature measurements during practical use.

The in-house prepared and calibrated TFTC was applied for the first time in the IGBT chip temperature-measurement field. By comparing the temperature-measurement data of the TFTC and the K-type wire thermocouple, the following findings were obtained: ➀ Due to its slightly smaller Seebeck coefficient, the temperature measured by the TFTC is relatively higher. ➁ Owing to their smaller heat capacity and rapid response, TFTCs exhibit higher heating and cooling rates compared to K-type wire thermocouples.

In the temperature-measurement experiments, variables such as the IGBT’s output current, the presence or absence of a radiator, the switching frequency, and the duty ratio of the control signal were set. It was discovered that the higher the output current, the higher the equilibrium temperature, as well as the higher the temperature-increase and temperature-decrease rates, which is consistent with the on-state loss formula. Adding a radiator can effectively reduce the equilibrium temperature, temperature-rising rate, and temperature-falling rate, thereby protecting the chip from drastic temperature changes, in line with Newton’s cooling equation. As the switching frequency increases, the equilibrium temperature, temperature-rise rate, and temperature-drop rate initially increase due to the growth in switching losses. Subsequently, as the switching time approaches the turn-on delay time, the output RMS decreases, leading to a decrease in temperature. Under certain frequency conditions, the higher the duty cycle of the control signal, the higher the equilibrium temperature, temperature-increase rate, and temperature-decrease rate. To sum up, NiCr and NiSi TFTC show great potential. In the actual experiment, the temperature measured by the TFTC is similar to that measured by the K-type wire thermocouple, and under the different experimental conditions of output current, heat sink addition or not, switching frequency and duty cycle of the control signal, the surface temperature of the IGBT can be measured stably and accurately, which helps to analyze the internal loss characteristics of the IGBT module. It can be regarded as a micron-scale thin-film temperature sensors, which will not damage the structure and physical characteristics of the tested element, and has the advantages of small volume, light weight, small heat capacity and fast response, which makes the TFTC have unique advantages in the field of temperature measurement of power devices.

To sum up, NiCr and NiSi TFTC show great potential. In the actual experiment, the temperature measured by the TFTC is similar to that measured by the K-type wire thermocouple, and under the different experimental conditions of output current, heat sink addition or not, switching frequency and duty cycle of the control signal, the surface temperature of the IGBT can be measured stably and accurately, which helps to analyze the internal loss characteristics of the IGBT module. It can be regarded as a micron-scale thin-film temperature sensors, which will not damage the structure and physical characteristics of the tested element, and has the advantages of small volume, light weight, small heat capacity and fast response, which makes the TFTC have unique advantages in the field of temperature measurement of power devices.

## Figures and Tables

**Table 1:** Comparison of different substrates.

Materials	Identity
Sapphire	Good heat resistance, impact resistance, but low strength, easy to be corroded by acid and alkali environment.
Ceramics	Better bonding, high melting point, high sensitivity and chemical stability, but the resistivity of ceramic thermoelectric materials tends to be larger, thus affecting the output of ceramic substrate thin-film thermocouple.
Polyimide	Transparent yellow, excellent thermal stability, chemical resistance, low coefficient of thermal expansion, high hygroscopicity.

**Table 2:** Plating parameters.

Test Parameters	Vacuum	Working Gas	Work Pressure/pa	Ar Rate of Flow/sccm	Splash Power/W	Splash Time/min
NiCr	6.0 × 10^−3^	Ar	0.6	20	150	18
NiSi	6.0 × 10^−3^	Ar	0.6	20	150	24

**Table 3:** Comparison of thin-film thermocouple and K-type wire thermocouple temperature measurement data.

Groups	Group 1	Group 2	Group 3	Group 4
Temperature at equilibrium measured by K-type wire thermocouples (°C)	42.80	31.32	80.59	53.13
Temperature at equilibrium measured by a thin-film thermocouple (°C)	45.12	33.65	87.03	59.52
Temperature difference between the two measured at system equilibrium (°C)	2.35	2.33	6.44	6.39
Maximum rate of temperature rise measured by K-type wire thermocouples (°C/S)	0.0757	0.0353	0.2001	0.0903
Maximum rate of temperature rise measured by a thin-film thermocouple (°C/S)	0.0778	0.0389	0.2253	0.1077
Absolute value of the maximum rate of temperature drop measured by K-type wire thermocouples (°C/S)	0.0464	0.0343	0.1138	0.0597
Absolute value of the maximum rate of temperature drop measured by a thin-film thermocouple (°C/S)	0.0505	0.0389	0.1327	0.0681

**Table 4:** Thin-film thermocouple temperature measurements with and without heat sink.

Groups	Load Carried 11.68 W	Load Carried 21.68 W
Equilibrium temperature with heat sink (°C)	33.65	59.52
Equilibrium temperature without heat sink (°C)	45.12	87.03
Temperature difference between the two caused by the heat sink (°C)	11.47	27.51
Maximum rate of temperature rise with heat sink (°C/S)	0.0389	0.1077
Maximum rate of temperature rise without heat sink (°C/S)	0.0778	0.2253
Maximum cooling rate with heat sink (°C/S)	0.0389	0.0681
Maximum cooling rate without Heat sink (°C/S)	0.0505	0.1327

**Table 5:** Thin-film thermocouple temperature measurement data at different output currents.

Groups	Without Heat Sink	With Heat Sink
The load carried is 11.68 W (°C)	45.12	33.65
The load carried is 21.68 W (°C)	87.03	59.52
Temperature difference due to different loads (°C)	41.91	25.87
Maximum rate of temperature rise measured with a load of 11.68 W (°C/S)	0.0778	0.0389
Maximum rate of temperature rise measured with a load of 21.68 W (°C/S)	0.2253	0.1077
Maximum cooling rate measured with a load of 11.68 W (°C/S)	0.0505	0.0389
Maximum cooling rate measured with a load of 21.68 W (°C/S)	0.1327	0.0681

**Table 6:** Thin-film thermocouple temperature measurement data at different switching frequency.

Groups	Switching Frequency 20 Hz	Switching Frequency 200 Hz	Switching Frequency 2000 Hz	Switching Frequency 5000 Hz
Equilibrium temperature (°C)	59.27	61.14	89.01	52.09
Maximum increasing rate (°C/S)	0.0972	0.1026	0.1735	0.0845
Maximum cooling rate (°C/S)	0.1509	0.1544	0.2822	0.1296

**Table 7:** Thin-film thermocouple temperature data for different output currents.

Groups	Duty Cycle 25%	Duty Cycle 50%	Duty Cycle 75%
Equilibrium temperature	40.72	60.98	80.06
Maximum increasing rate (°C/S)	0.0701	0.1272	0.1753
Maximum cooling rate (°C/S)	0.1017	0.1604	0.2378

## Data Availability

The original contributions presented in the study are included in the article, further inquiries can be directed to the corresponding author.
